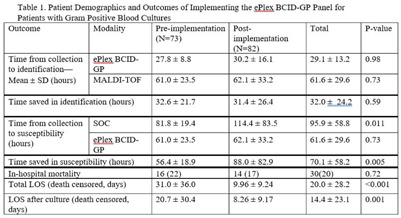# The impact of GenMark Dx ePlex blood-culture identification on the treatment and outcomes of gram-positive bacteremia

**DOI:** 10.1017/ash.2022.141

**Published:** 2022-05-16

**Authors:** B. Matthew Kiszla, Todd McCarty, Cameron White, Derek Moates, Sixto Leal, Rachael Lee

## Abstract

**Background:** In the treatment of bloodstream infections, the identification of the causal pathogen, and the evaluation of its susceptibility to antibiotics, often serve as the rate-limiting steps of the patient’s hospital stay. The GenMark Dx ePlex blood culture identification gram-positive (BCID-GP) panel aims to alleviate this bottleneck, thereby reducing the risk of severe complications and the spread of resistance, using electrowetting technology to detect the most common causes of GP bacteremia (20 targets) and 4 antimicrobial resistance (AMR) genes. We hypothesized that implementation of the ePlex BCID-GP panel would improve antimicrobial choice and de-escalation where appropriate. **Methods:** A mixed blinded and unblinded study was conducted to assess the effect of the BCID-GP panel on the outcomes and antibiotic stewardship of GP bacteremic patients before ePlex results were made clinically available (before implementation, N = 73) and once they accompanied the standard-of-care work-up (after implementation, N = 82). Differences in time to different benchmarks between the 2 modalities and the effect on patient outcomes were analyzed using null-hypothesis significance testing. **Results:** During the study, the BCID-GP panel identified 63 (42%) *Staphylococcus epidermidis* isolates, 31 (21%) *Staphylococcus* spp, 24 (16%) *Staphylococcus aureus* isolates, 12 (8%) *Streptococcus* spp, and 7 (5%) *Enterococcus* spp, and results were similar in the pre- and postimplementation groups (*P* = .13). The panel saved an average of 32.0 ± 24.2 hours in pathogen identification over standard-of-care methods, with no statistical difference made by the clinical availability of the data (Table 1). In terms of susceptibility testing, the panel saved an average of 70.1 ± 58.2 hours but with less unity between the 2 cohorts (*P* = .005). Of the 66 cases with follow-up, identification via ePlex indicated an escalation of therapy in 20 (30%) and a narrowing of coverage in 31 (47%). In patients identified to have *Staphylococcus aureus*, BCID-GP could change antimicrobial therapy in 79%; the need for escalation of antibiotics was identified in 58% of cases. In patients with *Staphylococcus epidermidis* bacteremia, implementation of BCID-GP panel could have resulted in de-escalation of antimicrobial therapy in 67% of patients. The implementation of the BCID-GP panel was correlated with no significant change of in-hospital mortality (*P* = .72) but was correlated with a significantly decreased death-censored total length of stay (LOS) (*P* < .001) and LOS after culture (*P* = .001). **Conclusions:** Our study has demonstrated that nonculture identification of bacteria and susceptibility can result in major improvements in antimicrobial therapy in patients, particularly those with contaminants identified.

**Funding:** GenMark DX

**Disclosures:** None

**Table:**